# The role of community engagement toward ensuring healthy lives: a case study of COVID-19 management in two Ghanaian municipalities

**DOI:** 10.3389/fpubh.2023.1213121

**Published:** 2024-01-18

**Authors:** Matilda Aberese-Ako, Phidelia Theresa Doegah, Lebene Kpodo, Wisdom Ebelin, Mawulom Kuatewo, Atubiga Alobit Baba, Atsu Godsway Kpordorlor, Samuel Yaw Lissah, Anthony Kolsabilik Kuug, Evelyn Ansah

**Affiliations:** ^1^Institute of Health Research, University of Health and Allied Sciences, Ho, Ghana; ^2^Pencils of Promise, Ho, Ghana; ^3^Evangelical Presbyterian Health Services, Evangelical Presbyterian Headquarters, Ho, Ghana; ^4^Hohoe Municipal Health Directorate, Ghana Health Service, Hohoe, Ghana; ^5^School of Public Health, University of Health and Allied Sciences, Ho, Ghana; ^6^Department of Hospitality and Tourism Management, Tamale Technical University, Tamale, Ghana; ^7^Department of Agricultural Sciences and Technology, Faculty of Applied Sciences and Technology, Ho Technical University, Volta Region, Ghana; ^8^School of Nursing and Midwifery, University of Health and Allied Sciences, Ho, Ghana

**Keywords:** Ghana, community engagement, sustainable development goal 3, COVID-19, community-based health planning and services, government institutions, healthy lives

## Abstract

**Introduction:**

Community engagement is one of the important requirements for strengthening health delivery in communities in a bid to achieve sustainable development goal 3, target 3.3 (SDG 3.3). The World Health Organization has strongly encouraged the use the five levels of community engagement, which are informing, consulting, planning, collaborating, and empowering communities in order to build resilience and to enable them contribute to the fight against diseases and for the uptake of health interventions. This study sought to explore and describe from the view of government institutions in Ghana how they engaged communities in COVID- 19 management and vaccine acceptance and how the communities within two municipalities also perceived the engagement process as well as the lessons that can be learned in engaging communities to deal with other health challenges and interventions toward the attainment of SDG 3 target 3.3.

**Materials and methods:**

This case study qualitative research project employed in-depth interviews among 36 respondents composed of government officials (the Ghana Health Service (GHS), the Information Services Department (ISD), the National Commission on Civic Education (NCCE) and two Municipal Assemblies), and community leaders and 10 focus group discussions among 87 men and women most of whom were natives and some migrants in two administrative municipalities in Ghana. Data were collected from June to September 2021. Audio interviews were transcribed and uploaded to Nvivo 12 to support triangulation, coding, and thematic analysis. Ethical approval was obtained from the University of Health and Allied Sciences’ Research Ethics Committee and all COVID-19 restrictions were observed.

**Results:**

The findings revealed that all the four government institutions educated and informed the communities within their municipalities on COVID-19 management and vaccine acceptance. However, the Ghana Health Service was the most effective in the engagement spectrum of the other four; consulting, involving, collaborating, and empowering communities in the process of COVID-19 management and vaccine acceptance. The GHS achieved that through its CHPS program, which ensured a decentralized health service provision system with multiple programs and leveraging on its multiple programs to reach out to the communities. Government institutions such as the NCCE and the ISD faced challenges such as limited funding and support from the government to be able to carry out their tasks. Additionally, they were not involved with the communities prior to the pandemic and for that matter, they did not have access to community systems such as committees, and existing groups to facilitate the engagement process.

**Discussion:**

Using communities to support Ghana’s attainment of the SDG 3 target 3.3 is possible; however, the government needs to provide funds and resources to the institutions responsible to enable them to carry out community engagement effectively. Also, promoting decentralization among institutions can strengthen community engagement processes. It is important that state institutions continue to strategize to empower communities in order to promote their participation in healthcare interventions and in the fight against infectious diseases in Ghana.

## 1 Introduction

Community engagement is essential for the delivery of primary health care and a people- centered care (1–4). This is because community engagement contributes to community buy-in to health interventions (5, 6), and ensures effective health advocacy and better healthcare service quality, which in turn influences clients’ satisfaction (7, 8). Effective community engagement enhances and increases the responsiveness of the healthcare system to clients’ health needs (9). Therefore, community engagement is essential for the effective implementation of health interventions such as those addressing communicable diseases, non-communicable diseases and maternal and child health among others (10–12). Nevertheless, existing evidence suggests that community engagement and intersectoral engagement have been the weakest link to primary health care since the Alma Ata declaration in 1978 (1). Thus, in order to achieve sustainable development goal 3: “Ensure healthy lives and promote well- being for all at all ages” (13), there is the need for a broader understanding of community engagement to support community empowerment to facilitate shared decision-making and to increase participation in the design and execution of health interventions (14–16).

Community engagement is useful for creating local and context-specific solutions for the prevention and response to health needs, which can facilitate the attainment of the set targets for sustainable development goal 3 (SDG 3) (13). The need for extensive community engagement in achieving SDG 3 target 3.3: “By 2030, end the epidemics of AIDS, tuberculosis, malaria, and neglected tropical diseases and combat hepatitis, water-borne diseases, and other communicable diseases” (13) is urgent and critical with the advent of pandemics such as Ebola and COVID-19. Documentation of experiences on community engagement with the advent of COVID-19 will help to inform future community engagement efforts in other areas of healthcare, which can contribute toward the attainment of the set targets of SDG 3.

This qualitative case study sought to explore and describe how the Ghanaian government through the Ghana Health Service (GHS) assisted by the, Information Services Department (ISD), National Commission on Civic Education (NCCE) and the Municipal Assemblies, engaged communities to support the fight against COVID-19 using the WHO’s community engagement framework. It also explored how the selected communities experienced the engagement process. Thus, the lessons from this study are useful for future policy-making and designing interventions toward Ghana’s quest to achieve the set targets of SDG3.

### 1.1 Community engagement defined

The World Health Organization (WHO), defines community engagement as: “…developing relationships that enable stakeholders to work together to address health-related issues and promote well-being to achieve positive health impact and outcomes” (17). The WHO’s (17) definition presents three main components, which are actors/stakeholders, developing the process, and the purpose of engagement. Stakeholders are perceived as the different parties that have an interest in the process, which comprises multiple communities that could include community members, patients, health professionals, policy-makers, and supporting sectors (18). The process suggests the different stakeholders strive to create a relationship that is based on respect, trust and a sense of purpose (17).

The framework proposes a spectrum of five components: (1) Informing the community of policy directions of the government; (2) Consulting the community as part of a process to develop government policy, or build community awareness and understanding; (3) Involving or planning with the community through a range of mechanisms to ensure that issues and concerns are understood and considered as part of the decision-making process; (4) Collaborating with the community by developing partnerships to formulate options and provide recommendations and; (5) Empowering the community to make decisions and to implement and manage change (19).

### 1.2 Context of community engagement in Ghana

One of the key approaches to the Ghanaian government’s fight against COVID-19 was to mandate key governmental agencies that are authorized to work with communities such as the District/Municipal Assemblies, the Information Services Department (ISD), National Commission for Civic Education (NCCE) and the Ghana Health Service (GHS), to engage with communities. The ISD, which is the oldest of the institutions was created prior to independence era and later transformed into the Department of Information. It serves as an effective unit for disseminating information from the Government. Specifically, to bridge the communication gap between the government and the governed (20). The NCCE on the other hand is aimed at creating awareness of government policies, programmes and activities through effective communication strategies using qualified human resources and state of the art technology to enhance national development, they also collate and assess public reaction to government policies and disseminate information on the activities of state officials and policies (21). The district assembly is aimed at decentralizing in order to ensure citizen participation by giving citizens the opportunity and power to engage in discussions and to contribute to decision- making processes affecting their districts. Consequently, citizen’s participation is pivotal to the decentralization programme of Ghana and such participation may involve information sharing, consultation, service access, election, and collaboration among others (22). Similarly, in the health sector, Ghana health service has made attempts to strengthen community engagement to ensure early and timely delivery of health services, and to improve the responsiveness of the healthcare system to curb widespread infections and reduce stress on the healthcare system in order to save lives (23, 24). As part of this measure, Ghana introduced the community-based health planning and services (CHPS) concept in 1997 with the following objectives: to strengthen health delivery through the mobilization of community leadership decision-making systems, and resources in a defined catchment area; the placement of reoriented frontline health staff with logistic support; and community volunteer systems to provide services according to the principles of primary health care (25, 26). Subsequently, the Ghana Health Service (government’s health service provision agency) and its partner organizations have established CHPS facilities in most districts to offer primary health care in communities.

## 2 Methods

### 2.1 Study design

The study used a case study qualitative approach to explore and describe the Ghanaian government’s community engagement efforts toward COVID-19 prevention, management, vaccine preparedness and acceptance in two municipalities in the Volta Region of Ghana. The study presented how these communities experienced the engagement process. Using in-depth interviews (IDIs) and focus group discussions (FGDs), the study explored in-depth knowledge on the Ghanaian government’s initiative through government institutions such as the Municipal Assembly, Ghana Health Service, Information Services Department, the National Commission on Civic Education and community experiences.

### 2.2 Study areas

The Volta Region was chosen for the study, because it recorded the lowest vaccine acceptance rate (32.50%) at the time of the study (27). Historically, the region has been observed by the Ghana Health Service as having the top 20 districts with the highest number of unimmunized children in the country, according to a 2021 report (28).

### 2.3 Selection of study sites

Two municipalities dubbed Municipality A* and Municipality B* were purposively selected for the study, because they are among the most urbanized municipalities in the region. The choice was aimed at understanding how urban populations engage with the healthcare system and other governmental entities in providing health interventions. Both municipalities have a fair proportion of the rural population and Municipality B also has migrant settlements mostly made up of persons from the Republic of Togo, with which it shares boundaries.

The Ghana Health Service operates a decentralized administrative system with offices at the national, regional and municipal levels, sub-district offices, hospitals, health centers, and CHPS facilities in each region (14). To ensure that the study was reflective of the different levels of health service delivery, a multi-stage random sampling technique was used to select a sub-municipality and a CHPS facility for the study in each of the two study municipalities. In the first stage, the names of all the sub-municipal health directorates in each study municipality were obtained from their respective Municipal Health Directorates. The names were written on pieces of paper, which were folded and an observer selected one sub- municipality for the study. In the second stage, all CHPS compounds under the selected sub- municipality were written on pieces of paper, which were folded and the observer randomly picked one. To ensure that community experiences were also captured in the study, a third stage was included, which concerned writing down the names of communities under each selected CHPS facility, folding it and letting the observer randomly select one to participate in the study.

### 2.4 Selection of study participants and sampling

From each of the study sites, one Municipal Health Service manager, one sub-municipal health service manager, two healthcare providers from the selected CHPS compounds, one official from each of following institutions: the Municipal Assembly, NCCE, and ISD respectively, and some of the community elders were purposively sampled to participate in IDIs (Table 1). A cross-section of women, men, and migrants of different age groups who were available and willing to participate in the study were conveniently sampled to participate in FGDs consisting of 6 to 13 participants. The interviews sought to understand how the Municipal Assembly, GHS, NCCE, and the ISD engaged communities in the COVID-19 vaccination rollout and uptake. Saturation was attained when no new information was obtained from study participants, which is in accordance with qualitative enquiry (29). Participation was voluntary and those who were not interested were automatically excluded as were those who were mentally challenged.

**Table 1:** **List of data collection methods and categories of respondents**.

Study participants who participated in IDIs	**Municipality A**	**Municipality B**
District Health Officials (1 municipal and 1 sub district)	2	2
Frontline workers in CHPS compounds	2	2
District Assembly officials	1	1
National commission for civic education	1	1
Information services department	1	1
Chiefs and queen mothers	2	2
Community Elders	2	2
Religious Leaders (Christian, Moslem, Traditionalist)		
Herbalists	2	2
Assembly persons	1	1
Community healthcare volunteers	2	2
**Total**	**18**	**18**
**Study participants for FGDs**		
Women below 30 years	8	8
Women above 30 years	10	8
Men below 30 years	9	9
Men above 30 years	9	8
*Migrant men	0	7
Migrant women	0	7
**Total**	**36**	**47**

*Municipality A does not have migrant settler communities; thus, no interviews were conducted for such a category.

The three components of the WHO’s definition of community engagement (categories of stakeholders, processes used to engage communities, and the purpose of the engagement), were used to guide the selection of the stakeholders, design of the IDI and FGD guides and to determine the focus of the study, which was on COVID-19 interventions (details of the questions for each spectrum of engagement has been included as Supplementary Appendix 1). This manuscript is drafted from a larger study and other aspects have been reported in another paper (30).

### 2.5 Data collection, quality control management and analysis

Four graduate data collectors were trained by the first author, MA, a medical and organizational anthropologist to conduct the interviews in the Ewe language for community members and in English for the government officials.

To ensure rigor, the study guides were pre-tested among eligible participants from a municipality similar to the municipalities selected for the study. The pre-testing process guided in the revision of the guides to ensure validity and reliability. In-person interviews were conducted in English with the government officials and in Ewe with the community members. Migrants were interviewed in French, as majority of them are from Togo and cannot speak English nor the Ewe dialect spoken in Ghana. Interviews were recorded using a digital audio recorder and later transcribed verbatim to preserve respondents’ views and experiences. The average duration of IDIs was 50 min and FGDs was 1 h. Meetings were held between MA and the data collectors every week to ensure the trustworthiness of data.

Transcribed data (IDIs and FGDs) were uploaded onto a computer and transferred onto a qualitative software NVivo 12, to support data coding. The data was triangulated and analyzed thematically. Deductive and inductive coding were carried out by LK, WE, AK and MK through carefully reading data, thinking critically and paying attention to the study questions, which were based on the five levels of community engagement (31). MA validated the codes by crosschecking them with the study questions and with a sample of study participants’ responses. Matrixes were developed from the coded data to support further analysis. The themes generated from the analysis report on how government institutions conducted engagement activities in the two study municipalities, challenges, gaps, future plans and community experiences. Face validity was established through reflections on the themes derived from the data during dissemination seminars at which the data was shared with local officials and community members in the two study districts. The participants confirmed that our study findings truly reflected their experiences. The drafting of the contents of this manuscript is guided by the “Consolidated criteria for Reporting Qualitative research” (COREQ) Checklist (32).

### 2.6 Ethics statement

The University of Health and Allied Sciences’ (UHAS) Research Ethics Committee (REC) approved the study [UHAS-REC A.5 (5 L 20–21)]. Potential study participants were approached and informed of the study and those who were willing to participate were taken through a consenting process. The consent form contained the following information: purpose, procedure, contact details of the principal investigator and the REC administrator, plan to disseminate study findings, date of consent, and signature columns for the interviewer and interviewee. Two copies of the consent form were completed, one was given to the study participant and the second copy was kept by the study team. Participation was voluntary and those who were not interested were automatically excluded, as well as those who were mentally challenged and persons under 18 years old. COVID-19 protocols were observed throughout the study. Interview participants were offered disposable masks and their hands were sanitized before consenting and participating in the interview. The data sets were anonymized to protect study communities and participants’ identities and were accessible to only the study team. Community entry was carried out in the study municipalities and study communities. The study team visited and sought permission from all gatekeepers (municipal chief executives, municipal health directors, CHPS compounds, officials of the NCE, and ISD, Assembly members, chiefs, and opinion leaders in the two municipalities).

## 3 Results

This section presents findings firstly, from government institutions on account of how they engaged communities and secondly, on how communities experienced and perceived the engagement process with these government institutions. Generally, findings from the IDIs and the FGDs suggest that all the four government institutions educated and informed the communities within their municipalities on COVID-19 management and vaccine acceptance. However, the Ghana Health Service was the most effective in the engagement spectrum of informing, consulting, involving, collaborating, and empowering communities in the process of COVID-19 management and vaccine acceptance. The GHS achieved this through its CHPS program, which ensured a decentralized health service provision system with multiple programs, which it leveraged to reach out to the communities.

### 3.1 Government officials’ engagement activities

The government workers from the four institutions (Ghana Health Service, Municipal Assemblies, the National Commission for Civic Education, and the Information Services Department) participated in IDIs and responded to questions concerning the levels of engagement such as informing, consulting, involving/planning, collaborating and empowering communities to make decisions on health issues.

#### 3.1.1 Informing communities about COVID-19

The government officials were asked questions about how they informed the communities in the catchment area on COVID-19 programs, the means they used to inform them, the processes used, the languages used among others. Findings from the IDIs revealed that all the four institutions informed the communities in their catchment area and educated them on COVID- 19 and vaccine acceptance. The institutions used channels such as the radio, information vans and existing community information systems. Additionally, the institutions used community gatekeepers such as elders, chiefs, and assemblymen. All the institutions utilized flyers and communicated mostly in the native language (Ewe). The GHS’ CHPS facilities used routine health care programs like immunization services, child welfare clinics (CWC) and community groups that they formed prior to the advent of COVID-19, including mother-to-mother support groups among others, to reach out to communities.

##### 3.1.1.1 Ghana health service

The study participants revealed that the GHS used the CHPS facilities as a major avenue to engage with communities. The CHPS compounds, which are located close to or within the communities, built relationships with communities over time. Additionally, the CHPS facilities initiated the formation of community groups prior to the advent of COVID-19 such as community health volunteers, mother-to-mother support and men support groups, which they leveraged to inform communities about COVID-19 management and vaccine acceptance. Also, the Regional Health Directorate (this is the administrative wing of the GHS at the regional level) encouraged the CHPS facilities to form community health management committees on COVID-19 prevention and management and vaccine campaign groups to provide information and education on COVID-19 to the communities. Furthermore, IDIs with health workers and health managers revealed that the Ghana Health Service informed the communities within their jurisdiction about COVID-19 prevention, management, and vaccine acceptance using the following methods: community durbars to educate them on COVID-19 in the indigenous language (Ewe) and using social gatherings such as church activities, funerals in the communities as a platform to inform and educate them on COVID- 19. Another platform that the CHPS facilities used was community gatekeepers such as chiefs, queen mothers, and elders, whom they held meetings with to inform them, and they in turn passed the messages to their communities. They also used routine health programs and health delivery access points such as antenatal clinics, post-natal clinics, child welfare clinics (CWC), and outpatient department platforms to inform and educate the community members. The CHPS facilities with the support of the District Health Directorates designed COVID-19 messages and used community resources such as the local community radio, the Community Information Centers, and beating gong-gong [local drums] to disseminate COVID-19 messages to the communities. They also used durbars to educate the communities and to encourage them to take vaccines. A health manager reported that this initiative influenced the community members to vaccinate “We went to areas that we have identified as places which need to be vaccinated. So, we selected those places. And when it came, we went round educating them. Luckily for us, the people participated well during the first round of vaccination.” (Health Manager, Municipality A).

##### 3.1.1.2 Municipal assemblies

Municipality A and Municipality B assemblies reported that they designated the NCCE and the ISD to inform and educate the communities on COVID-19. The NCCE indicated that they met the chiefs prior to the visits to the communities to seek their permission to carry out the education. Other strategies included educating community focal persons and unit committee members (unit committee is the lowest level of the decentralized district assembly system, which is based in communities) and gatekeepers such as chiefs, elders and religious leaders to use their privileged positions to educate the community members. They also utilized community resources such as community information centres and radio stations to inform the communities. They printed leaflets containing COVID-19 preventive information. In addition, the assemblies communicated in the English and Ewe languages to communities in order to ensure that all the members of the communities understood the messages.

##### 3.1.1.3 The national commission for civic education

The National Commission for Civic Education (NCCE) in the two municipalities revealed that due to the ban on social gatherings they were meeting the communities in segments, based on the existing associations and the various traditional and social groupings. They met the chiefs prior to the visits to the communities to seek their permission to carry out the education. They broadcasted COVID-19 education messages at dawn and dusk in order to reach the entire community. They used the community information system like the GHS and the municipal assemblies to inform the communities of COVID-19 management and vaccine acceptance.

However, the NCCE reported that the community members were not enthused with the education on COVID-19 and it did not influence their behavior either. They also admitted that though their van was old they were able to put it to good use by mounting a megaphone speaker on it. They also indicated that they are able to visit the community to educate them about prevention and vaccine acceptance as an interview participant shared the experience: “We organize the people through the Assembly member and the chiefs, so they beat gong for the people to come out. We talked to them about the pandemic. We have educated them on the vaccine as well but they did not take it kindly with us.” (NCCE Official, Municipality A).

##### 3.1.1.4 The information services department

The ISDs in the two municipalities reported that they used existing radio stations to broadcast information in the Ewe language to the communities and they also used public places such as the market square. There was a collaborated effort between the ISD in Municipality B and the two other government institutions - GHS and NCCE as they reported that they used the jingles prepared by the GHS to educate the communities in the local language. They further teamed up with the NCCE and the GHS to visit the communities because it was exceedingly difficult for them to reach all the communities within the municipality. as the van assigned to them had broken down. Other hurdles faced by Municipality B included community members demanding money from them and misconceptions about the COVID-19 vaccines, which they put in a lot of effort to convince the communities that it is good.

#### 3.1.2 Consulting

The government officials were asked questions that centered on how the feedback or opinion of the communities on the different occasions was included in proposals on COVID-19 programs, how the feedback from the communities was included or used to implement COVID- 19 programs, and how the communities were provided with feedback on how their inputs influenced the COVID-19 programs that the government institutions undertook.

##### 3.1.2.1 Ghana health service on consulting

Study participants from the GHS indicated that they consulted the communities, took their views on proposals in their programs on COVID-19 management and vaccine acceptance, and implemented those they deemed appropriate. A municipal health manager cited an instance when the Ghana Health Service requested that every household should acquire a Veronica bucket for handwashing but various communities suggested using tippy taps as an alternative because they could not afford the cost of the Veronica bucket and it was widely accepted. Additionally, ISD also offered the communities the opportunity to communicate their proposals to them whenever they wish. The ISD offices in the two municipalities reported that they consulted the communities on COVID-19 issues, but their primary responsibility was to disseminate information and update the municipal assembly on the communities’ concerns. They indicated that it is the municipal assembly’s responsibility to incorporate the community’s concerns, so they often conveyed community concerns to the assembly, which in turn incorporated such concerns into their plans.

“Usually, when we meet with them, we get instant feedback. But we also make an allowance that should they have enough information to give to us, they can contact us to give it to us through the community health committee members.” (Health Manager, Municipality A).

“So, when we take their views, then we see how to integrate it into whatever we are doing. So, we see how best to integrate whatever they want, not what we want, but then we see to it that it is the best for them and us.” (Nurse, Municipality B CHPS facility) However, a health manager in one of the sub-district health facilities in Municipality A said that the facility had never received any suggestion from the community for consideration since the emergence of COVID-19 (Assistant Health Manager, Municipality A).

##### 3.1.2.2 The municipal assemblies on consulting

The two Municipal Assemblies consulted with the communities and groups on how to stop the spread of COVID-19, and suggestions made by the community members were taken into account and implemented. For instance, Municipality A created satellite markets and changed the market days as requested by community members to reduce the congestion at the central market to reduce the risk of transmission. Municipality B also included the needs of the community in the municipal assembly’s medium- and long-term plans. An official shared the interactions with the communities and the various proposals that the communities made to the assembly:

“Yes, we do [accept community proposals]. For instance, somewhere last year [2020], we even selected some businesses from various communities and brought them here to solicit their views…some of these proposals are long-term, and others are medium- term. So, we have incorporated them into a plan. …For instance, one of their major challenges was opening of the border. The Assembly has tried to communicate that to the government. The second one is the provision of water…The Assembly has committed its funds to drill a mechanized borehole for the people so that they can practice hygiene…Yes, we could not go back to the community and do the announcement as we used to do in terms of coordinating.” (Assembly official, Municipality B).

##### 3.1.2.3 National commission for civic education on consulting

The findings also reveal that although the NCCE in Municipality A consulted the community in addressing COVID-19 management and vaccination issues, they did not immediately address the proposals given by communities at the municipal level; instead, they sent communities’ suggestions to the national headquarters of the NCCE for consideration. They added that some of the proposals that were health-related were referred to the GHS for consideration. In contrast, the NCCE office in Municipality B said that they had not consulted any of the communities within the municipality, because the majority of them did not believe that COVID-19 exists.

##### 3.1.2.4 The information services department on consulting

Additionally, ISD also offered the communities the opportunity to communicate their proposals to them whenever they wish. The ISD offices in the two municipalities reported that they consulted the communities on COVID-19 issues, but their primary responsibility was to disseminate information and update the municipal assembly on the communities’ concerns. They indicated that it is the municipal assembly’s responsibility to incorporate the community’s concerns, so they often conveyed community concerns to the assembly, which in turn incorporated such concerns into their plans.

#### 3.1.3 Planning/involving

Study participants from government institutions were asked how they had been working with communities in taking their views in planning programs on COVID-19 and how they had been guiding them to propose alternative interventions on COVID-19.

##### 3.1.3.1 Ghana health service on planning

All the Ghana Health Service facilities reported that they planned COVID-19 activities with the community members. They used channels such as community durbars, Child Welfare Clinics, and Parent-Teacher Association meetings. During such occasions, community members’ views were sought on how to prevent COVID-19 and how to successfully roll out the COVID-19 vaccines.

Additionally, a CHPS facility in Municipality A reported that it had reactivated health committees before the advent of COVID-19, and these meet every 3 months. So, they were leveraging on it to have regular stakeholder consultations with community gatekeepers and institutional heads in planning on COVID-19 management and vaccine acceptance. A nurse from a CHPS facility in Municipality B also reported that they engaged the community for planning purposes by first contacting the Community Health Management Committee (CHMC), which is a committee that was created at the community level to support the CHPS facilities. Also, health officials reported that they had developed relationships with the communities and earned their trust by participating in community activities such as funerals and community meetings. Such interactions helped communities trust that the health workers cared about them, which always made them willing to plan with the health workers toward the delivery of health interventions.

“We make sure that we engage them, communicate the message to them to understand, take time to address every concern that they have, so we will have a shared common vision.” (Health Manager, Municipality A).

“Because of that good collaboration between the community and the health facility, any time we approach him [community chief], he normally listens to us. So, in terms of the planning towards COVID-19, we plan together.” (Nurse, Municipality B CHPS facility).

##### 3.1.3.2 The municipal Assembly’s experiences in planning with communities

The findings also revealed that both Municipality A and B assemblies included community opinions when planning COVID-19 events and activities by seeking the opinion of community gatekeepers such as chiefs, opinion leaders, and assembly members. Also, leaders of community groups and associations such as market women groups, drivers’ unions, motorbike riders’ associations, and small-scale business associations in the municipalities were involved in planning and decision-making on COVID-19 interventions. They reported that with such constant engagement, the various stakeholders listen to them and implement the recommended directives.

“When we engage them, we also listen to them and we give them the message on how to prevent the spread of the disease. …For example, we met the drivers, we told them to always make sure everybody is in a nose mask before getting in their vehicles. So if you are not in a nose mask, they insist that you buy one around and put it on before joining their vehicle” (Assembly Official, Municipality A).

##### 3.1.3.3 National Commission for civic education on planning

Additionally, the findings revealed that the NCCE in Municipality A usually allowed the communities to give them feedback after informing and educating them to help them plan future activities. “…With the communities, when we go, after our education, we ask them to tell us their understanding and they should tell us what they feel we should do better next time.” (NCCE official, Municipality A) The municipality B NCCE on the other hand reported that they only notified the Chiefs and the Assemblymen whenever they wanted to have programs including COVID-19 programs in the communities, however, the communities were not involved in the planning of health intervention activities including COVID-19.

##### 3.1.3.4 Information services department on planning

The information services department of Municipality A and B reported that they obtained feedback on community needs at community durbars after they had presented their information.

An ISD official from Municipality B cited an instance when Municipality B Assembly presented sanitizers and face masks to communities to facilitate COVID-19 observance after the NCCE had informed the assembly of such a request from the community.

#### 3.1.4 Collaborating

Study participants from the government institutions were asked how they had been working with the communities in their catchment area to find possible solutions to fighting COVID-19 and how they had been seeking community advice on COVID-19 programs.

##### 3.1.4.1 Ghana health service on collaboration

Study participants from the GHS indicated that the different channels that they used to engage the communities had helped to create partnerships between them and the communities because it had enabled the communities to present their views, some of which had been implemented. Instances where the GHS received feedback from communities on languages to communicate in and the need for communities to improvise the use of Veronica buckets by using tippy taps created from local materials were cited. Also, a community donated COVID-19 prevention items to a health facility to enable them to observe the protocols at the facility.

The Ghana health service reported that they had been seeking community advice through meetings with elders and soliciting their help. A nurse from a CHPS facility in Municipality A cited an instance when they sought the help of a community under their catchment area for help and they referred them to reach people within the community who could help the health facility.

“We have committee members that we have been working with, so to get solutions for solving these problems, we let them point out those in the community who can help us to enable us to help the community. So, they …showed us the people and we contacted them to plead with them to help us …. So, they provided the things that we needed such as soap, water, hand sanitizer, and other materials.” (Nurse, Municipality A CHPS facility).

The GHS participants in the two municipalities reported extensive collaborative relationships with the communities in their catchment area over the years. They leveraged such collaboration for interventions concerning COVID-19. The health managers at the Municipal level ensured that the CHPS facilities used the following strategies: community meetings, regular calls to communities to update them on important health activities and diseases, and through a community-based surveillance volunteer group. Municipality B cited examples that it was through the management committee’s insistence that they had to create a treatment center in the municipality for COVID-19 patients and also extended the language for informing communities to Ewe and English.

“Through the community members, the community meetings, and the reviews that we do. In Municipality B, we have a policy. Every month, even if you are a CHPS compound, call your community members and tell them the pattern of diseases that you are finding. Tell them the number of people you immunized in your catchment area, tell them the number of people who come to the hospital, and the type of diseases they are bringing to the hospital… We have these Community-based Surveillance Volunteers who also report on community events. So, we use that feedback from the community to tailor education and the way we go about our work.” (Health Manager, Municipality B).

“We also have Public Health Emergency Management Committee meetings which include the chiefs and we have been having these Public Health Emergency Management Committee meetings every two weeks. So, it is also a means of giving feedback to the communities and the chiefs also contribute at that Public Health Emergency Management Committee meeting. So, it is more of a debriefing on whatever that is happening and they also give advice and we implement it.” (Health Manager, Municipality B).

The two GHS authorities in the two municipalities reported that the collaboration had contributed to an effective flow of information between the GHS and the communities, which culminated in trust and a supportive relationship. A sub-district manager cited an instance of a community leader supporting one of the health facilities, when they asked for help to enable the facility to practice the COVID-19 protocols is reported as follows:

“We do this through durbars. We realized that some of the facilities do not have veronica buckets like my own for instance. So, during the durbar, we made it known to the community and then they also came out with the view that some of them can supply and …the assemblyman for this particular community, donated the veronica bucket to us with some toilet rolls.” (Sub District Manager, Municipality A).

A few health officials reported that they had not sought the communities’ views, as they perceived them as ignorant, so they carry out education to the communities most of the time. However, one official admitted that they had not made efforts to seek the community’s experience:

“If I say I do seek their advice, I would say I am telling lies, because most of the time, I do the telling….: Yes, I do the education…. We the health professionals especially in this sub-district usually do the talking and the people seem to be ignorant, but I think I already have one problem which is we usually do n’ot ask them much about their opinion.” (Sub District manager, Municipality A).

##### 3.1.4.2 Municipal assembly on collaboration

The findings also suggest that Municipality A assembly usually, worked together with the community to receive views from community members, however, some of the views were not implementable. They cited two instances, the first where a community proposed that they used a local plant to treat COVID-19, which they could not accept, because it had not been medically proven to be efficacious. In the second instance, the Municipality A assembly reported that the community supported them in identifying a facility for admitting persons who got infected with COVID-19. Municipality B assembly on the other hand, reported that they were unable to go down to the community to receive their feedback, because they were being cautious due to the spread of the disease.

“During the engagement with the community, we always bring issues before them for their inputs. So, for example, when it came to where to host people infected with the COVID-19, we had to engage the community. So that is when the idea came that there is one abandoned building somewhere in town, which can be put to that use. So that idea came from the community. So, we went to visit the place and we saw that the place is a bit isolated. If the assembly can put a few touches to it, that place can be useful. And it has worked on well.” (Assembly official, Municipality A).

##### 3.1.4.3 NCCE on collaboration

The NCCE of Municipality B said that their role was solely to educate communities. The NCCE.

in Municipality A reported that it had education clubs in schools, which provided them with reports on activities in the schools. The Municipality A NCCE indicated that they usually met with the gatekeepers such as chiefs who present the communities’ suggestions to them for further action.

“In some of the communities, we have our patrons over there. We also have our civic education clubs in the schools. So, we get reports from them. We also have the community child protection committees. Also, Plan International came in to provide a lot of logistics and other personal protective equipment (PPEs). So, after the community engagement or before we go, we interact with the opinion leaders such as the chiefs, the community protection officers, and other notable people in the community. So, we all sit down and discuss whatever they tell us. So, that’s what we normally do so that it would improve our next line of action.” (NCCE official, Municipality A).

“We are to go to educate them. The possible solution is education, to change their mindset about the pandemic. That is what we are doing.” (NCCE Official, Municipality B).

The findings also indicated that the experience of the NCCE is not different from the GHS and the district assemblies. Whilst Municipality A reported that they encouraged the communities to give them feedback on their activities, which were usually incorporated into NCCE’s activities,. Municipality B reported that they did not seek advice or feedback from their communities.

“We ask them to tell us what they think about the way we are performing our duties. We ask them to tell us what they think about the education, the dawn broadcast, the meetings, and the seminars.” (NCCE Official, Municipality A).

##### 3.1.4.4 ISD’s Activities on collaborating with communities

The ISD study participants in the two municipalities indicated that the community members usually gave them feedback after they provided them with information at community meetings and community suggestions that concern health issues to the Municipal Health Directorate for them to act and other general needs to the municipal assemblies that are responsible for such communities. They indicated that because they lacked the resources to respond to communities’ health needs, they focus on informing communities communicating their needs to institutions that are mandated or have the resources to address those needs. The ISDy also indicated that some of the feedback that they got from the community was wrong perceptions, which they corrected.

#### 3.1.5 How the government institutions empowered the communities

Questions on empowerment that were posed to government officials were ways in which government institutions equipped the communities with knowledge on COVID-19, how government institutions supported the communities to identify resources that can be used to support their fight against COVID-19, and how the government institutions had equipped them to take their own decisions on the fight against COVID-19. Out of the four institutions that were interviewed, only the GHS reported that they had been able to empower the communities, the municipal assemblies, the ISD, and the NCCE did not have an instance where they had been able to empower communities.

##### 3.1.5.1 Ghana health service’s experiences in empowering communities

Majority of the GHS facilities in the two study municipalities empowered the various communities under their catchment area on the prevention and management of COVID-19. They recommended to the communities to improvise Veronica buckets by using old gallons to make tippy taps and place them at vantage points in all households for hand washing. They also encouraged them to sew face masks by using old cloths, educated them to cover their nose and mouth when coughing as well as on the proper usage of nose masks. Other activities were proper hand washing with soap under running water, the use of hand sanitizers, adherence to social distancing and reporting to health authorities of any suspected case exhibiting COVID-19 signs and symptoms. However, some of the Ghana Health Service facilities reported that they did not empower the community members to use their resources in the fight against COVID-19.

“We did not tell them how to use their resources to support the COVID-19 fight.” (Nurse, Municipality A CHPS facility).

“I would not say there is much support from my end to identify the resources that can be used to fight COVID-19.” (Health Center in-charge, Municipality B).

### 3.2 How communities experienced engagement

Study participants such as gatekeepers and a cross-section of community members from 18 years and above shared their experiences on how they were informed, consulted, involved, or participated in planning, collaborated with, and empowered to take decisions on COVID-19.

#### 3.2.1 Informing communities on COVID-19 management and vaccination

The findings revealed that the government institutions informed communities about COVID- 19 prevention, as most of the study participants indicated that they were educated on safety protocols. They went ahead to provide accurate information about the safety protocols. However, only a few respondents provided some accurate information about the COVID-19 vaccines and mentioned GHS as the only government institution, which informed them about the vaccine.

##### 3.2.1.1 Informing and educating communities on COVID-19 preventive measures

Findings from the FGDs and the IDIs from the community members showed that the GHS and the NCCE educated them on the COVID-19 safety protocols such as wearing nose masks, using sanitizers, hand washing, and avoiding handshakes and crowded places. They reported that both institutions adopted multiple strategies in providing information to them. The GHS they reported made use of more diverse strategies as compared to the NCCE in informing and educating communities. The GHS used community durbars, social gatherings, home visits, routine health programs, and the media, while the NCCE used the media and announcement vans.

“… they [nurses] call us to the roadside and meet with the whole community and the chiefs and tell us about the protocols such as washing of hands, avoiding shaking of hands… They tell us all the time that when we adhere to those protocols the disease will be prevented.” (Herbalist, IDI, Community A).

Few of the study participants stated that the nurses [GHS staff] also employed the home visit strategy. The nurses from the CHPS facilities and community health volunteers visited the homes of community members to educate them on the COVID-19 safety protocols.

“They [nurses] visit us in our houses and tell us the things we should do such as how to wash hands. So, some time ago I had a hand washing facility in my house, but currently it is no longer here.” (Herbalist, IDI, Community A).

The nurses at the CHPS facilities also used social gatherings such as funerals, schools, and churches to inform the community members about COVID-19. They visited these gatherings, educated the attendees on COVID-19 prevention, and taught them to observe the protocols. Study participants shared their experiences. FGD participants reported that the nurses who worked in the CHPS compounds took advantage of routine healthcare programs such as Child Welfare Clinic (CWC) sessions to educate the women on COVID-19 prevention. A study participant shared her experiences:

“They talk to us about the disease [COVID-19] when we come for weighing too.” (Female FGD Participant, Community B).

The study participants reported in IDIs and FGDs that the COVID-19 pandemic had made it impossible for community leaders to hold meetings with government stakeholders. Consequently, the GHS and NCCE used the mass media, specifically, radio to inform them about COVID-19 prevention. Most community members identified the GHS and the NCCE as the main source of education on the radio.

“Most times like the district aspect, when you switch on your radio, you will hear that the people are from the health directorate and that they will be talking about COVID- 19. So, they will do education on COVID-19 for instance, how we should wash our hands, how we should protect ourselves with the mask and everything.” (Church Elder, IDI, Community B).

“Anytime we tune to the radio, they [NCCE officials] talk on it and teach about how the COVID-19 disease is and how we can take care of ourselves so that we do not get the disease.” (Female FGD Participant 30 years or less, Community B).

Study participants confirmed that the NCCE also used vehicles fitted with public address systems to educate communities in Ewe, on how they can protect themselves from COVID-19.

“… They [NCCE] come with their vans and they talk about health issues, how to keep ourselves safe from the disease [COVID-19].” (Assemblyman, IDI, Community A).

“They (NCCE) use a van with a public address system at the top and they use Ewe [indigenous language] to make the announcements.” (Male FGD Participant above 30 years, Community B).

##### 3.2.1.2 Informing communities about COVID-19 vaccination

The findings from the IDIs and FGDs with study participants from the communities revealed that education on the COVID-19 vaccination at the time of the study was not intense. When asked what the participants knew about the COVID-19 vaccine roll-out and the source of information, only a few of the respondents in the study communities reported that they were informed by GHS about COVID-19 vaccines and vaccine rollout. They further reported that the health workers in the CHPS compounds informed them about the vaccine. The CHPS facilities also used routine healthcare programs such as child welfare clinics (CWC), where women send their children for weighing and medical checkup to inform mothers of COVID- 19 vaccination. Additionally, GHS staff used interpersonal communication such as informing community members in their neighborhood about the COVID-19 vaccine.

“For the CHPS compound staff, they asked them to make us aware of the vaccine and they did. So, they told us about plans for the vaccination. Since the vaccine has not arrived here yet, it was just the announcement that they made to us.” (Herbalist, IDI, Community A).

“Our nurses told us at the weighing (CWC) that they will be coming to administer the vaccine.” (Female FGD Participant above 30 years, Community B).

A few of the study participants reported that they had not been informed about getting vaccinated. Some attributed the failure of the various government agencies to inform them of the vaccine to the fact that the available vaccines were meant for only top government officials and essential workers such as health workers. They expressed trust and confidence in the health workers, that whenever the vaccines became available, the health officials will vaccinate them.

“The CHPS compound workers are not telling us anything, because the vaccine has not come to us yet. The vaccine has not come to the community yet, so they cannot tell us that they will vaccinate us or they will not vaccinate us. They are just waiting for the government to see whether they will bring it. We are also just waiting for them to see whether they will bring it. When they come, the CHPS compound will let us know about them.” (Chief, IDI, Community A).

##### 3.2.1.3 Planning or involving communities on COVID-19 management and vaccination

Interactions with gatekeepers and a cross-section of study participants from the communities revealed that a few of them were involved in the planning of COVID-19 programs. The study participants gave specific examples on occasions that the government institutions solicited their views or ideas in planning for COVID-19 prevention programs.

“The one I can mention is, when they came to talk to us about the wearing of face masks, we agreed on using the cloth ones and they immediately went ahead to advise us to be using the cloth ones. So, I can say that our opinion was taken.” (Female FGD Participant above 30 years, Community B).

“In planning for programs, they meet and seek advice on how they should plan. Then the nurses too will tell us their thoughts …, when they realize that whatever we tell them is good, they accept it. But if they do not agree to what we say, they also suggest or give their views for us to consider.” (Male Community Health Volunteer, IDI, Community B).

Some of the community members however indicated that they did not participate in the planning meetings. Several gatekeepers who participated in the IDIs as well as the community members who participated in the FGDs reported that they were not involved in planning of health programs. They explained that the health workers brought their plans to implement and, on some occasions, they only sought clarification to be able to support the implementation process.

“When we meet, whatever they tell us to do, we only tell them how we think we can go about it. We do not tell them our ideas. We do not bring any ideas. They bring the idea and we tell them how we understand the idea.” (Community Elder, IDI, Community A).

“They do not receive any other views from us than the nurses’ thoughts which they implement. When they want to do some work in the community, they gather us and announce for us to come to the roadside and they explain the thing to us. When an.

aspect is not clear to us, we also ask them and they will explain to us before they start the work.” (Female FGD Participant above 30 years, Community A).

“They do not take plans from us. They make their plans and bring them to us. I do not remember having a meeting with them to make plans. The plans always come from their end to us.” (Male, FGD Participant 30 years and less, Community B).

##### 3.2.1.4 Consulting communities on COVID-19 management and vaccination

The views of all the study participants such as gatekeepers and a cross section of community members who participated in the study, were sought on how they were consulted on health programs including COVID-19. Some of them revealed that the government institutions consulted them and incorporated their views in the implementation of programs. The study participants cited instances when they chose to use face masks and it was accepted as a proof that the government institutions take their opinions into consideration.

“I would say they took our opinion into consideration because, when we agreed on using the cloth masks, that was what was made readily available for us.” (Female FGD Participant, 30 years or less, Community B).

“Time after time, …the feedback we have given them or the proposals we have made to them, they go and modify their programs and come to us again with new strategies in combating the coronavirus.” (Assemblyman, IDI, Community A).

“When we meet and we share our ideas with them, they use the ideas to implement activities for us to see.” (Female FGD Participant, 30 years or less, Community A).

Other study participants noted that some of their views were accepted if they did not involve the use of resources. Nevertheless, community views that concerned interventions that had a cost element were not implemented.

“So they accept it but those that involve money, they leave it down for the community. They expect the community itself to do it. But those that demand ideas, they accept it gladly, because there will not be any financial commitment.” (Community Elder, IDI, Community B).

Other study participants admitted that they shared their views but they indicated that they could not confirm whether their views were included in the implementation of programs, since the health workers did not confirm to them whether they were included.

Some of the study participants reported that their ideas were not included in the implementation of COVID-19 programs. Others confirmed that they had been having meetings and they had been making proposals, however, they were not sure whether their ideas were being incorporated because the government officials did not usually give them feedback.

“… we have been having meetings and they have been writing minutes [government officials] but we do not know if they include it into their proposals. They do not tell us whether the discussions that we have been having are included in their works.” (IDI with Chief, Community B).

“They do not take our opinions; I guess they feel they know what is best for us.” (Male FGD Participant, above 30 years, Community A).

##### 3.2.1.5 Collaborating with communities On COVID-19 management and vaccination

Some of the gatekeepers and the other study participants reported that the government institutions particularly, the GHS collaborated with them in addressing health problems including in fighting COVID-19. They reported that the CHPS facilities worked with the community health volunteers and also used the existing community health committee to discuss their plans and only implement them after the committee has given them permission.

“There is a committee for the CHPS. So, they do not do things on their own. Before anything, they meet the committee first and inform them. The committee also discusses it before giving permission to them to carry on.” (IDI, Female Community Health Volunteer, Community A).

“Whenever they face any challenges, they inform the volunteer. Then we also meet as a committee and meet the need.” (IDI, Traditionalist, Community A).

“When they are done with the directives they brought us, where the directive comes from, they make us aware of it. When we decide on issues, they come to us with the solutions and tell us what to do and inform us that they have accepted our suggestions.” (IDI, Queen Mother, Community B).

Nonetheless, some community gatekeepers reported that there was no collaboration between them and the government institutions. They indicated that the government institutions have not created opportunities for them to meet them to offer advice for health programs.

“I cannot say anything about that because for now they are not yet working with me to find a solution for COVID-19. They have not called us to any workshop on COVID 19 yet.” (IDI, Male Community Health Volunteer, Community A).

“With that one, they do not seek for our advice.” (IDI, Community Elder, Community A).

##### 3.2.1.6 Empowerment of communities on COVID-19 management and vaccination

Some of the study participants indicated that the information that the government institutions provided to communities on COVID-19 empowered them to take initiatives to prevent contracting the disease. However, others reported that they did not feel empowered enough to take the COVID-19 vaccine.

###### 3.2.1.6.1 Adherence to COVID-19 safety protocols

Study participants reported in the IDIs and FGDs that the information that they received from the government institutions empowered them to make decisions on their own to safeguard their health by adhering to the safety protocols without being forced. They indicated that they practiced physical distancing, hand washing, wearing face masks, and using hand sanitizers. They indicated that their main source of empowerment was from the health workers, particularly those at the CHPS facilities.

“…because they are the nurses, we have to accept whatever they tell us. So … since the disease came and the health workers at the CHPS compound told us to wear nose masks and wash our hands, we have been doing it on our own…” (IDI, Queen Mother, Community B).

“Through their [the government institutions] teachings and training, we realized that if we do not comply with the preventive measures, we will contract the infection. So… we are able to make effective decisions to stay safe and healthy.” (Male FGD Participant 30 years and less, Community B).

Study participants revealed that they applied the knowledge they gained from the education that they received from the government institutions by using local, easily accessible, and affordable materials to make their own handwashing stations (tippy taps and soap containers) and to sew face masks.

“First, we did not know how to make a tippy tap for hand washing, but through them [the nurses], we know how to do it and when we go out for a while and come back home, we wash our hands or if we have sanitizers, we use them. So, I have realized that it is helping us to also prevent the virus.” (IDI, Community Health Volunteer, Community B).

“The people at the CHPS compound told us that even if we cannot get the big veronica bucket, we can perforate some containers and use them. As I earlier said, you can put a rope around the container, and by pulling the rope, water comes out for you to wash your hands. They said we should wash our hands under running water… When they first came with the message, everybody went ahead to do a hand washing facility in his or her house. Everybody was washing their hands under running water. They placed soap beside the handwashing facility. It means we all understood it.” (IDI, Male Community Health Volunteer, Community A).

Study participants reported that some of the community members who were seamstresses and tailors voluntarily sewed nose masks and distributed them to other community members free of charge, while others took advantage of the situation to sew masks for sale. Also, the youth in the community organized themselves to make face masks and distribute them for free among community members.

“Actually, we have a section of our youth who also saw the need to help fight against this deadly coronavirus. They educate us on community support. So, sections of the youth also go into making nose masks. So, they have made a lot of nose masks and distributed them to every member of the community. I took part in distributing those nose masks.” (IDI, Assemblyman, Community A).

“I said earlier that, when the disease was detected, our tailors used their own clothes to make face masks and some people said even though we have been hit by a disease, some people got an opportunity to make some money.” (Male FGD Participant above 30 years, Community B).

Some community leaders reported that they were empowered by government agencies to take the initiative of educating their communities. They used social and religious gatherings to sensitize community members on COVID-19 prevention measures and to encourage them to observe them.

“When we go into a gathering, we ourselves are able to announce that, the rules that the government has made, everybody should endeavor to obey.” (Chief, IDI, Community A).

“There are dangers involved if we do not obey their (health workers in CHPS facilities) advice. Their advice has made us to involve ourselves and give information to one another… As pastors or church leaders, we have been tasked that at least we should use 5 minutes to sensitize or to educate the congregants or the church members on COVID-19… Almost every Sunday we do it. Even if something happens and we are not able to do it before service starts, in the middle or in the midst of the service, we do it. So that is part of the empowerment.” (IDI, Pastor, Community A).

##### 3.2.1.7 Influence of community engagement on intention to get vaccinated.

Majority of the study participants reported that they had not been given ample information on COVID-19 vaccines, to enable them to make a firm decision to get vaccinated, so they were not willing to take the vaccine. They explained that the government institutions did not provide ample information for them concerning the vaccines as they did for the COVID-19 preventive measures. Others also indicated that while they were happy to observe the protocols, they were not willing to take the vaccine.

“Concerning that vaccine, in fact, some of us will not accept the vaccine. Honestly, we say we are not receiving it. I can encourage people if I hear further clarification from the health institutions and I understand. Only then that I can encourage someone to also take it, because I also understand something and can explain to the person that he should also take it. But as they came just like that without giving us information about it, even though some of us are working with the health facility, we are scared.” (IDI, Health Volunteer, Community B).

“…I will not take the vaccine. I just do not want to take it. I will only take it if they give me detailed education on it.” (IDI, Queen mother, Community B).

Despite the information provided to community members by the government institutions, some of the community members who participated in the study were unwilling to vaccinate.

“Please I would say we should continue observing the safety protocols that we have been taught. Taking the vaccine is not an option for me. If something should kill me, it should not be a vaccine.” (FGD participant, female, above 30 years, Community A).

“I will never take it, let alone my children. They can come and kill me; I will not take it.” (Male Migrant FGD participant, Community B).

“When it comes and it turns out that I have the virus, when they give me the vaccine, I will accept it. …nobody will take it because there is no virus here. When you bring it to them, they will all say no! no! no!” (IDI, Fetish priest, Community B).

A few of the respondents were willing to take the vaccine. Their decision was based on the little information they had received from the government institutions and their trust in the government. Study participants believed that the vaccine was safe, it will prevent them from contracting COVID-19. Additionally, participants believed that the government will not set out to deliberately harm citizens by bringing a poisonous substance to inject them.

“Oh yes, I will take the vaccine. They said [government officials] …that when I take the vaccine, I will be free and it will prevent the disease from attacking me, that is why people are taking the vaccine.” (Chief, IDI, Community B).

“Yes! That is what I have already said, the government cannot buy any infected vaccine to use to kill those under him. So, for me when they come, I will agree and go for the jab. Because it is clear to me.” (Chief, IDI, Community A).

## 4 Discussion

The desire to achieve the SDG 3, which is anchored on good health and well-being of people, requires the full participation of community members in order to enhance a desirable health program implementation leading to its achievement. The 17 SDGs are integrated; they recognize that action in one area will affect outcomes in others and that development must balance social, health and environmental sustainability. In line with this, the study sought out the views of how community engagement could promote COVID-19 prevention and vaccine acceptance in Ghana and how this can serve as lessons for other infectious diseases. The study explored how communities experienced engagement using the WHO community engagement model in a low resource setting. The study also examined the influence of community engagement on intention to get vaccinated. The findings suggest that the Ghana Health Service was the most effective regarding the levels of community engagement such as informing, consulting, involving, collaborating, and empowering communities in the process of COVID- 19 management and its vaccine acceptance. The GHS achieved this through its CHPS program, which has enabled a decentralized health service provision system with multiple programs and a highly interactive system at the community level (22, 33). Similarly, other studies in Ghana have noted the influence of the CHPS program in community involvement resulting in effective implementation of healthcare interventions (33, 34). This is important to note considering that community engagement is the foundation of the CHPS programme (33). The CHPS initiative is Ghana’s flagship strategy for achieving universal health coverage (UHC) and if it continues its community engagement effort especially in rural communities it will play an important role in Ghana’s attainment of SDG 3 (35–37).

Interactions with study participants revealed that many community members adopted the COVID-19 prevention protocols (hand washing, distancing, masking), however, they were hesitant in getting the vaccine to protect them from severe COVID-19. Similar strategies were used by all the 16 regions and the world over in dealing with the COVID-19 prevention, however, there was no uniform strategy in promoting the vaccine, which could have affected willingness to vaccinate. Because, the initial vaccine consignment that the country received was meant for a select few, so, there was no uniform message provided to communities. Some of the different institutions adopted either a ‘wait and see’ approach and others went ahead to inform the public but did not provide the vaccine. Others also provided opinion leaders the first dose but delayed in providing the second dose for vaccines that required two shots (30).

Anecdotally, individuals willingly accepted the preventive measures because they perceived the measures will not have any effect in their body, whilst in relation to the vaccine, individuals had trust issues regarding its perceived negative effects on them. Essentially, because of the rapid development of the vaccine (30, 34). This finding is not surprising considering that historically, the region has been observed by the Ghana Health Service as having the lowest rate of vaccine acceptance with the top 20 districts with the highest number of unimmunized children in the country (28). Additionally, it recorded the lowest vaccine acceptance rate (32.50%) in 2020 (27). An earlier paper from the current study reported that the causes for vaccine hesitancy included challenges such as insufficient logistics and myths and misconceptions about vaccines, which accounted for some community members’ lack of trust in vaccines, resulting in their unwillingness to vaccinate (30). Also, Kuatewo et al. (34) have noted that in addition to the challenges mentioned, a popular song in the local language in the Volta Region encouraging community members not to vaccinate contributed to vaccine hesitancy.

Majority of the study participants reported that they had not been given ample information on COVID-19 vaccines, to enable them to make a firm decision to get vaccinated, so they were not willing to accept the vaccines. They explained that the government institutions did not provide enough and accurate information for them concerning the vaccines as they did for the preventive measures. This finding from the study contrasts with another study (38), where it was found that, in low- and middle-income countries, community engagement has been a critical enabler of effective responses to controlling communicable diseases (38). Similarly, community engagement was effective in responding to the 2014 Ebola outbreaks in the context of a weak healthcare system in Sierra Leone, where community response teams were instrumental in interrupting the local transmission through contact tracing, house-to- house visits, health facility and community reporting (21, 39).

The findings suggest that, government workers from the four institutions that participated in the study informed the communities in their municipality and educated them on COVID-19 and vaccine acceptance. This finding corroborates other studies, which found that community engagement is crucial for reaching marginalized people and promoting their participation in health and other social interventions (11, 12). This current study also compares with other studies on community engagement concerning experiences from outbreaks, which show that community engagement can take many forms and include different actors and approaches to prevention and control activities, including designing and planning, community entry and trust building, social and behaviour change communication, risk communication, surveillance and tracing and logistics and administration (6, 21, 40). Nonetheless, all these studies including the current one point to the effectiveness of using community engagement to promote acceptance of social interventions such as vaccine acceptance, which could lead to good population health. And once the health of the people is ensured, it would contribute toward the attainment of the set targets for SDG 3.

The study found that most of the time the NCCE, ISD and the district assembly rarely gave communities feedback on how their suggestions had influenced the COVID-19 programs that they were running. Such an approach contributed to distrust of government institutions and the void created led to misinformation resulting in mistrust, which contributed to vaccine hesitancy. Similarly, other studies have noted that community engagement has been limited to informing communities or engagement between government institutions with limited involvement of communities (19). Limited involvement of communities and ‘top-down’ approaches used within COVID-19 responses may not yield the needed vaccine acceptance, since they are likely to mistrust the intervention, resulting in hesitancy in the uptake of COVID- 19 vaccines and other health interventions (3, 4). Studies elsewhere have noted that there is a gap between community needs and how public services are rendered, because of the high tendency toward a top-down approach to community engagement (16, 41).

The findings revealed that the Ghana Health Service gave feedback to communities on their suggestions and contributions. Consequently, most of the community respondents trusted the GHS more than the other government institutions. Similarly, a study carried out by Islam et al. (42), found that community engagement in the form of feedback and involving residents led to successful management of the COVID-19 outbreak. Baltzell et al.’s (6) study found that the best strategy to ensure effective community engagement is to make it interactive between the community and institutions. Such a strategy will contribute to communities believing that their issues are important and will whip their interest in public interventions and help to forge lasting partnership.

Most of the Ghana Health Service facilities reported that they planned COVID-19 activities with the community members. Some of the channels used were gatherings such as community durbars, Child Welfare Clinics, and Parent-Teacher Association meetings. During such occasions, community members’ views were sought on how to prevent COVID-19 and how to successfully roll out COVID-19 vaccines. This study’s finding corroborates with studies carried out by Atkinson et al. (11) and Baltzell et al. (6), which found empowering communities to plan intervention activities played a role in successful disease control and elimination campaigns in many countries. The similarity in terms of the findings could be a result of the fact that health officials had developed a familial relationship with the community members and earned their trust by participating in community activities such as funerals and community meetings. Community engagement has been noted to help build trust in the healthcare system, which is crucial for effective healthcare delivery and clients’ satisfaction (6, 36, 40). Community engagement serves as a platform for building community trust in the healthcare system and other government institutions responsible for providing social services. Thus, emphasizing community engagement is as crucial for community participation in activities toward achieving SDG 3.

In the area of collaboration, study participants from mostly the GHS’ CHPS facilities indicated that they used different channels to create partnerships between them and the communities. It enabled the communities to present their views and some of those views were taken up and those that were not feasible were not taken. This contributed to the observance of the COVID-19 protocols in the communities. Additionally, by collaborating with the communities, it enabled the community leaders and the different community groups to educate their members on the protocols, which contributed to sustaining the gains and ensuring that even when the institutions were not policing the communities, they still observed the protocols. This finding agrees with Ogundijo et al.’s (43) and Decouttere et al.’s (41) studies, that community engagement is a viable way for monitoring the spread of SARS-CoV-2 and ensuring effective collaboration. This is not new; experiences from fighting other infectious diseases such as malaria and Ebola revealed that collaboration between relevant authorities and communities contributes to good coverage and effective implementation of interventions (3, 17). The finding also provides lessons on how to ensure sustainability of intervention projects and that investing in collaboration is cost effective. Additionally, this finding indicates that achieving the SDG3 requires partnership with other agencies or sectors.

The study found that majority of the CHPS facilities within the GHS in the two study municipalities empowered the communities under their catchment area on the prevention and management of COVID-19 by training them to make their own soap locally for handwashing and making nose masks using pieces of cloth. This ensured that communities were able to take decisions that they were comfortable with and independently. Community engagement that empowers communities and utilizes community resources can contribute largely to the effective implementation of social and health interventions as well as the fight against pandemics (6, 41). Similarly, Head (44) has noted the importance of building the capacity of communities to engage effectively. In achieving the SDG3, this finding shows the need to engage in capacity building activities at community levels to ensure an empowered population. The desire to achieve the SDG 3, which is anchored on the good health and well-being of people, requires the full participation of community members in order to enhance a desirable health program implementation leading to its achievement. The 17 SDGs are integrated; they recognize that action in one area will affect outcomes in others and that development must balance social, health and environmental sustainability. In line with this, the study sought out the views of how community engagement could promote COVID-19 prevention and vaccine acceptance in Ghana and how this can serve as lessons for other infectious diseases. The study explored how communities experienced engagement using the WHO community engagement model in a low resource setting. The study also examined the influence of community engagement on intention to get vaccinated.

### 4.1 Strengths and weaknesses of the study

This study had some limitations and strengths. It was carried out prior to the country receiving adequate vaccines for community members and this made it difficult to have a complete understanding of community response to vaccination. A second limitation of the study is as it is typical in qualitative research, a limited number of government officials, community gatekeepers, and a cross-section of community members participated in the study, which does not enable generalization. Nevertheless, by interviewing different categories of community members and government officials and triangulating the data from the different sources, this study was able to bring out extensive views and experiences of the different stakeholders in community engagement. This study’s strength lies in the fact that the nature of COVID-19 poses ethical dilemma, making person-to-person contact for such a study was complex and yet it was carried out successfully, thus, contributing literature to the very few qualitative in-person studies that have been carried out to understand government and community experiences in dealing with pandemics. Another strength of the study is that it is the first, as far as we are aware, to use the WHO community engagement framework to examine or assess COVID-19 management and vaccine acceptance using a qualitative approach.

## 5 Conclusion

This case study used the WHO’s community engagement framework to describe how government institutions addressed COVID-19 management and vaccine acceptance in a Ghanaian context and the lessons that can be learned to facilitate the attainment of SDG 3 by 2030. The findings suggest that the GHS was able to maintain the most sustainable and effective way of engaging communities through its CHPS program. Also, the multiple programs at the CHPS level, the activities of Municipal Assemblies, and the Information Service Department facilitated the development of the different levels of engagement.

The findings further suggest that community engagement in the fight against infectious diseases toward attaining the set targets of SDG 3 in Ghana is feasible using the current system. Nevertheless, to make the current system effective, the government needs to empower and resource the different government agencies to enable them to carry out effective engagement exercises.

Additionally, to be able to sustain engagement processes, government institutions need a continued and long-term relationship with the community and this requires that the various institutions maintain continuous interactions with communities and not an on-and-off relationship. Our findings also suggest that the management of pandemics such as COVID-19 can be enhanced by the effective involvement of community members in preventive strategies and feedback mechanisms.

Even though the findings suggest that the current system is effective in curbing the transmissibility of infectious diseases, more needs to be done regarding the provision of factual and accurate information, to communities whilst dispelling misconceptions that impede the prevention of infections through vaccines.

Policy makers need to factor in community engagement as a core component of policy interventions when designing policies, as policy is only as good as its implementation is accepted by beneficiaries. Thus, making room for resources to be channeled toward community engagement in any social intervention is important for success.

The healthcare system needs to sustain the gains made over the years in community engagement processes. Nevertheless, it was noted that some of the healthcare providers did not practice all the levels of engagement, which compromised the quality of the information, especially on vaccination. It is recommended that community engagement efforts should be strengthened within the healthcare system. Also, the other institutions need to emulate the GHS by using the existing community structures and systems to carry out community engagement to support the rollout or scale-up of interventions.

The Volta Region remains the region historically suspicious of vaccines and low in acceptance of healthcare interventions especially vaccination. It is important that special attention is given to the region in engagement processes to ensure that more gains are made, if Ghana is to achieve the set targets of SDG 3.3.

We recommend that more studies on community engagement should be done on other healthcare interventions in order to contribute to literature and policy. Exploring the dynamics of community engagement beyond COVID-19 to other interventions will be useful in contributing to knowledge and future policy.

## Data availability statement

The raw data supporting the conclusions of this article will be made available by the authors, without undue reservation.

## Ethics statement

The studies involving humans were approved by the University of Health and Allied Sciences’ Research Ethics Committee [UHAS-REC A.5 (5 L 20–21)]. The studies were conducted in accordance with the local legislation and institutional requirements. The participants provided their written informed consent to participate in this study.

## Author contributions

MA-A, LK, PD, and EW contributed equally to the paper. MA-A and EA conceptualized the study. MA-A trained LK, EW, AK, and MK to collect, code and analyze the data. EW, MK, LK, and AGK collected, coded, and analyzed the data. MA-A and PD supervised the data collection. MA-A, LK, PD, and EW drafted the manuscript. MK, LK, AKK, AB, SL, and EA reviewed the manuscript critically. All authors contributed to the article and approved the submitted version.
