# Supplementary material for: The role of community engagement toward ensuring healthy lives: a case study of COVID-19 management in two Ghanaian municipalities
Source: Front Public Health. 2024 Jan 18;11:1213121. doi: 10.3389/fpubh.2023.1213121 (PMC10832024; doi:10.3389/fpubh.2023.1213121)
Supplement: Supplementary file 1 [file Data_Sheet_1.PDF]

IN-DEPTH INTERVIEW GUIDE ON "ENGAGING COMMUNITIES IN RESPONDING TO COVID-19 IN LOW RESOURCE COUNTRIES: A CASE STUDY OF GHANA"

Religious (Christian, Moslem, Traditional Leaders), chiefs, queen mothers, youth leaders, migrant community leaders

Section A: Background characteristics

Community, name, position in community, age

Section B: History on government-community engagement on health

1. How have you and the community been involved by the following government institutions: GHS (CHPS compound in the community, sub-district health directorate etc.), the National Commission for Civic Education (NCCE), the Information Services Department (ISD) in healthcare programmes in the last ten years? (Pick each institution and probe the follow-up questions)
2. Tell me about how you and the community have been working with (mention each government body) to deal with health issues in this community.
3. How long have you and the community been working with them on health care programmes and how did it start?
4. Kindly mention some of the healthcare programmes that you have worked with them in the past?

Section C: Government- Community engagement on COVID-19

I would like to ask you questions about how you and the entire community have been engaged in the entire process of information, prevention and vaccination preparedness

1. For a year now a new disease has been declared a pandemic, which is called COVID-19. What have you heard about it?
    a. Where did you hear about it?
2. Which government institutions have been working with you and the community on COVID-19 issues? GHS (CHPS compound, sub-district), NCCE, ISD? (Pick each institution and probe the subsequent questions)

3. **Informing**
    a. How are you informed (institution) on COVID-19 programmes in your community and the district? (means of informing, processes, language used)
    b. Since the advent of COVID-19, how many times has (mention institution) informed you and the community about government programmes on COVID-19?
    c. How did you understand their messages and how detailed/comprehensive was it for you?
    d. Were you encouraged to ask questions and were they addressed?
4. **Planning/involving**
    a. How have (institution) been working with you and the community to take your views in planning programmes on COVID-19?
    b. How have (institution) been working with you and the community in guiding you to propose alternative interventions on COVID-19?
5. **Consulting**
    a. How was your feedback/opinion on the different occasions included in proposals for COVID-19 programmes?
    b. How was your feedback included or used to implement COVID-19 programmes?

   c. Kindly tell me about how (mention institution) provided you and the community with feedback on how your inputs influenced the COVID-19 programmes that they are undertaking.

6. **Collaborating with the community by developing partnerships to formulate options and provide recommendations.**
   a. How has (mention institution) been seeking community advice on health programmes?
   b. How has (mention institution) been working with you and the community to find possible solutions to fighting COVID-19?
7. **Empowering the community to make decisions and to implement and manage change**
   a. In what ways has (institution) equipped you and the community with knowledge on healthcare issues?
   b. How has it supported you and the community to identify resources that can be used to support your fight against COVID-19?
   c. How has (institution) equipped you and the community to take your own decisions in the fight against COVID-19?

Section D: Persons involved in the engagement process

1. What has been your role in the engagement process?
2. Who are the other community members who have been involved in the engagement process?
   a. Probe for religious (Christian, Moslem, Traditional Leaders), chiefs, queen mothers, youth leaders, migrant community leaders, ordinary community members
3. How are they engaged by the government bodies (mention each)? (Probe for meetings, frequency, information provided at meetings)
4. How useful has (institution) engagement process been for you and the community? (Probe for trust in government information, sense of ownership, influence in reaching a broad range of community members, empowering them to take key health care decisions)

Section D: Innovative strategies to community engagement

1. How do you think the community can be better engaged in the healthcare programmes?
2. How should the government institutions go about engaging the community?
3. What are the existing community resources that can be used to support the engagement process?
4. How can you and the community support the government to improve the engagement process in this community (inform, consult, involve, collaborate and empower)?
5. Any other recommendation will be very much welcomed.

Section F: Community engagement and vaccine acceptance

1. Please, what do you know about the COVID-19 vaccines?
2. Tell me about your knowledge on Ghana's vaccine rollout?
   a. What is your source of information?
3. Tell me about the community's concerns with the vaccine?
4. What are your concerns about the COVID-19 vaccine?
   a. Probe: Why?
5. How has (institution) engaged you and the community in the vaccine rollout? (engagement spectrum)

6. After (the institution) has engaged you, what concerns do you and the community still have about the vaccine? (exclude this question if engagement process is done)
7. How do you think community engagement can help address these concerns?
8. How do you think the government bodies can improve community engagement process to ensure vaccine acceptance?
    a. Probe how he/she should be involved?
    b. How can the community support the process?
9. Will you be willing to take a COVID-19 vaccine?
    a. Probe: why
10. Will you encourage others to take the COVID-19 vaccine?
    a. Probe: why

11. What other issues do you want to share on COVID-19 and the vaccination?

Thank you!
